# Prognostic value of unrelated atypical serum immunofixation patterns during Multiple Myeloma therapy

**DOI:** 10.1186/1756-8722-5-33

**Published:** 2012-06-26

**Authors:** Cristina Guimarães, Rui Bergantim, Renata Ramalho, Nuno Couto, João T Guimarães, Fernanda Trigo

**Affiliations:** 1 Department of Immunology, Faculty of Medicine, University of Porto, Alameda Professor Hernâni Monteiro, 4200, Porto, Portugal; 2 Department of Hematology, Centro Hospitalar São João, Porto, Portugal; 3 Department of Medical Oncology, Portuguese Institute of Oncology Francisco Gentil, Porto, Portugal; 4 Department of Clinical Pathology, Centro Hospitalar São João, Porto, Portugal, Portugal

**Keywords:** Multiple myeloma, Autologous stem cell transplantation, Marker of prognosis, Serum immunofixation patterns

## Abstract

Autologous stem cell transplantation (ASCT) is the gold standard therapy for suitable multiple myeloma (MM) patients after induction with high dose therapy. To date, the evidence of a reliable marker of prognosis in these cases remains scarce. Our aim was to evaluate appearance of unrelated atypical serum immunofixation patterns (ASIPs) as a marker of prognosis in MM patients submitted to ASCT. We retrospectively analysed data from 65 patients. Interestingly, we observed that presence of ASIPs was associated with longer progression-free survival and longer overall survival. Our results suggested that presence of ASIPs could be a novel marker of good prognosis in MM patients submitted to ASCT.

## Letter to the editor

Multiple myeloma (MM) is a plasma cell neoplasia characterized by abnormal production of monoclonal immunoglobulin detectable in serum and/or urine [1]. However, no reliable markers for prognosis in MM patients submitted to autologous stem cell transplantation (ASCT) are available. The appearance of unrelated atypical serum immunofixation patterns (ASIPs) is a well-recognized event after ASCT in MM with a prevalence ranging from 10 % to 73 % [2-4] and a marked reduction of the malignant plasma cell clone in presence of ASIPs has been described [5]. Interpretation of ASIPs appearance during MM therapy have been challenging for clinicians [6]. Although the real value of ASIPs after ASCT remains controversial, it might be an undervalued surrogate marker for prognosis.

Our aim was to evaluate the appearance of ASIPs on serum immunofixation (IEF) as a prognosis marker in MM. Patients with MM, less than 65 years old and without comorbidities that underwent ASCT were included. Staging of disease followed the Salmon-Durie and International Staging System (ISS) and number of ASCT per patient was recorded. Immunotypes were determined by IEF (HYDRASYS® agarose gel electrophoresis). ASIPs was defined as the appearance of new mono or oligoclonal immunoglobulin protein bands (with either light or heavy chain components). Patients were stratified according to the presence or absence of ASIPs. Overall survival (OS) and progression-free survival (PFS) were calculated using Kaplan-Mayer method and comparisons between groups were made using log-rank test. Chi-square test for categorical variables and Kruskal-Wallis test for continuous variables were used. Analysis of Covariance (ANCOVA) was performed. A *p* value of 0.05 was considered significant.

Between January 2000 and June 2009, 65 patients with MM submitted to ASCT were studied (Table 1). According to the serum IEF analysis, 42 patients had presence of ASIPs after ASCT. There were no significant differences in number of ASCT, Salmon-Durie and ISS stages in patients with presence/absence of ASIPs. However, these groups differed in age (58 ± 5 years for presence of ASIPs versus 54 ± 7 years for absence of ASIPs, *p* = 0.012) and in death rate (31.0 % for presence of ASIPs versus 60.9 % for absence of ASIPs, *p* = 0.019).

**Table 1:** Clinical parameters after ASCT according to presence or absence of ASIPs on serum IEF

**Characteristics**	**Presence of ASIPs** **(n = 42)**	**Absence of ASIPs** **(n = 23)**
Age at dx (years)	58 ± 5	54 ± 7^a^
ASCT		
1 ASCT (n) 2 ASCT (n)	11 (26.2 %) 31 (73.8 %)	11 (47.8 %) 12 (52.2 %)
Salmon-Durie (n)		
IA	5 (11.9 %)	0
IIA	7 (16.7 %)	5 (21.7 %)
IIIA	23 (54.8 %)	13 (56.5 %)
IIB	2 (4.8 %)	1 (4.3 %)
IIIB	5 (11.9 %)	4 (17.4 %)
ISS (n)		
I	8 (19.0 %)	3 (13.0 %)
II	22 (52.4 %)	15 (65.2 %)
III	12 (28.6 %)	5 (21.7 %)
Relapses (n)	29 (69.0 %)	18 (78.3 %)
Deaths (n)	13 (31.0 %)	14 (60.9 %)^b^

DX – Diagnosis, ASCT – Autologous stem cell transplantation, ASIPs – atypical serum immunofixation patterns, ISS – International Staging System.^a^P = 0.012, Kruskal-Wallis test; ^b^P = 0.019, chi-square test.

**Figure 1:**
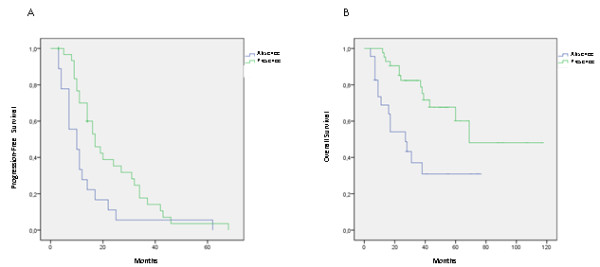
**Patients with MM submitted to ASCT with presence of ASIPs had longer progression- free survival (A) and overall survival (B) (*p*** **= 0.014 and*p*** **= 0.002).**

Oligoclonality, as compared to the absence of ASIPs, was associated with a hazard ratio for relapse of 0.49 (95 % CI, 0.26-0.89; *p* = 0.019). Figure 1A shows the PFS in the two groups. Median PFS was 13.1 months (for patients with absence of ASIPs (95 % CI, 6.8-19.4), and 22.6 months for patients with presence of ASIPs (95 % CI, 17.2-27.9; *p* = 0.014). As compared to the absence of ASIPs, the presence of this component in serum IEF was also associated with a hazard ratio for death of 0.33 (95 % CI, 0.15-0.69; *p* = 0.004). Figure 1B shows the OS in these two groups. Median OS was 35.6 months (95 % CI, 23.1-48.1) for patients with absence of ASIPs, and 78.1 months (95 % CI, 61.2-95.1; *p* = 0.002) for patients with presence of ASIPs. When adjusted for age, PFS and OS remained significantly different between the two groups (*p* = 0.009 and *p* = 0.014, General Linear Model approach to ANCOVA). These results points toward the hypothesis that presence of ASIPs may be a protector feature in MM patients submitted to ASCT. This is in concordance with recent results from Wadhera and colleagues [7].

We concluded that presence of unrelated atypical serum immunofixation patterns could be a possible laboratory marker of good prognosis in MM patients after ACST. Further studies are needed to confirm our results.

## Abbreviations

MM, Multiple myeloma; ASCT, Autologous stem cell transplantation; IEF, serum immunofixation; ASIPs, atypical serum immunofixation patterns; ISS, International Staging System; OS, overall survival, PFS: Progression-free survival.

## Competing interest

The authors declare that they have no competing interests.

## Authors’ contributions

CG and RR designed the study; CG, RR and RB interpreted data; GC and RR wrote the manuscript; RB and FT reviewed diagnoses; RR and NC performed the statistical analysis; RB and FT collected data. All authors have read and approved the final manuscript.
